# Diagnostic yield and number needed to screen for cervical and concomitant spinal injuries in geriatric head trauma

**DOI:** 10.1016/j.bas.2026.106247

**Published:** 2026-07-27

**Authors:** Josina Straub, Helena Arias, Melanie Ardelt, Jonas Krueckel, Hannah Pielmeier, Manuela Malsy, Gerardo Napodano, Borys Frankewycz, Daniel Popp, Maximilian Kerschbaum, Volker Alt, Siegmund Lang

**Affiliations:** a Clinic and Policlinic for Trauma Surgery, University Hospital Regensburg, Germany; b Department of Orthopaedics and Traumatology, University Hospital Krems – NOE LGA, Karl Landsteiner University, Mitterweg 10, Krems, 3500, Austria; c University for Continuing Education, Danube University Krems, Krems, Austria; d Clinic for Anesthesiology, University Hospital Regensburg, Germany; e Department for Radiology, University Hospital Regensburg, Germany; f Department of Clinical Acute and Emergency Medicine, University Hospital Regensburg, Germany

**Keywords:** Geriatric patients, Cervical spine injury, Concomitant spinal injury, Risk factors, csMRI, Number needed to screen

## Abstract

**Introduction:**

Geriatric patients with head trauma face an increased risk of cervical spine injuries (CSI) and concomitant spinal injuries (CoSI). The added value of cervical spine MRI (csMRI) and whole-spine imaging alongside routine cranial CT (cCT) and cervical spine CT remains unclear.

**Research question:**

This study evaluates the prevalence and risk factors for CSI, the number needed to screen (NNS) to detect CoSI and the diagnostic yield of csMRI.

**Materials and methods:**

Patients aged ≥65 years presenting after head trauma to a Level I trauma center between January 2020 and October 2025, undergoing cCT and cervical spine CT, were analyzed.

**Results:**

A total of 2748 geriatric patients were included, with 63.9% aged ≥80 years and 1.5% showing sensomotoric deficits. CSI occurred in 6.0%, equally affecting axial and subaxial regions. Conservative treatment of CSI was employed in 75.2%, while dorsal instrumentation was the most performed operative technique (63.4%). Risk factors for CSI included advanced age (OR = 1.1, p = 0.03), ankylosing spinal disease (OR = 5.9, p < 0.001) and thoracic trauma (OR = 2.9, p < 0.001). The NNS for incremental yield of csMRI was 54.9 for patients aged ≥65 years and 45.3 for those aged ≥80 years. For CoSI the NNS were 29.4 and 19.3 respectively. Among patients undergoing additional csMRI, subaxial injuries were observed in 45.6%, with 70.0% being treated conservatively.

**Discussion and conclusion:**

CSI are common in geriatric head trauma and frequently accompanied by CoSI. Given the low NNS, a liberal use of extended imaging, including whole-spine CT and selective csMRI, can be considered to improve patient safety.

**Level of evidence:**

III, survey.

## Highlights

• Cervical spine injuries are common after geriatric head trauma.
• Concomitant spinal injuries frequently accompany cervical spine trauma.
• CsMRI shows limited additional diagnostic yield after initial CT.
• Whole-spine CT detects clinically occult injuries with low NNS.
• Extended imaging should be considered in selected geriatric patients.

## 1 Introduction

Projections by the United Nations Department of Economic and Social Affairs indicate that by 2050, one in five people worldwide will be aged 65 years or older, a demographic shift accompanied by increasing comorbidities, frailty and polypharmacy, collectively heightening vulnerability and the risk of adverse health events, including falls (Shi et al., 2023; Prabhakaran et al., 2020). Annually, approximately one-third of individuals aged ≥65 years’ experience at least one fall, with about half of these incidents requiring emergency medical care (de Wit et al., 2020). In the United States, this corresponds to roughly 2.8 million older adults presenting to emergency departments for fall related injuries, representing 10 to 15% of all emergency department visits (Galet et al., 2018; Sethi et al., 2014). Fall related injuries, predominantly involving head and extremity trauma, further result in an estimated 800,000 hospital admissions each year (Bergen, 2016; Engelbart et al., 2022).

In geriatrics, cervical spine injuries (CSI) can occur even from low-energy events such as ground-level falls (Jeanmonod and Varacallo, 2025). Regarding to literature, more than 60% of geriatric CSIs are caused by falls, with at least half resulting from ground-level incidents (Golob et al., 2008). These injuries most commonly involve the upper cervical spine (Sethi et al., 2014). The prevalence of CSI among older adults reflects increased susceptibility to low-impact falls and injuries often extend beyond a single vertebral level and are commonly accompanied by additional fractures, underscoring the profound skeletal vulnerability associated with conditions such as osteoporosis and ankylosing spinal disorders (Malik et al., 2008; Shimizu et al., 2025; Delcourt et al., 2015). In addition, recent nationwide data demonstrated that advanced age, concomitant injuries, and comorbidities substantially contribute to in-hospital mortality in patients with cervical spinal cord injuries (Ardelt et al., 2025). Computed tomography (CT) is considered the premier diagnostic modality for the precise identification of cervical spine fractures (Inaba et al., 2016). In geriatric patients presenting after fall-related trauma, clinicians bear the responsibility of carefully determining the need for imaging, including a thorough assessment of the cervical spine (Engelbart et al., 2022). Two widely recognized clinical decision instruments for guiding the use of cervical spine CT are the Canadian C-spine rule and the National Emergency X-Radiography Utilization Study (NEXUS) criteria (Stiell et al., 2001; Goode et al., 2014). The Canadian C-spine rule, however, specifically excludes patients aged 65 years or older from clinical clearance, limiting its applicability in the geriatric population (Goode et al., 2014). Although the NEXUS criteria have been employed in geriatrics, reported sensitivities in this group vary, reflecting potential limitations of the clinical examination, particularly in patients with cognitive impairment such as dementia (Hoffman et al., 2000; Paykin et al., 2018). Consequently, current guidelines advise neither Canadian C-spine rule nor NEXUS should be solely relied upon for clinical decision-making in patients aged 55 years or older (Gramlich et al., 2020; Tran et al., 2016). When evaluating geriatric patients with minor trauma, clinicians are often entrusted with the responsibility of relying on their own clinical judgement, all while confronting the significant financial and resource implications of radiologic investigations (Tran et al., 2016; Jambhekar et al., 2018).

In patients with CSI, concomitant trauma to other body regions is common, affecting approximately 60% of cases (Miller et al., 2011; Anandasivam et al., 2021). Studies employing whole spine imaging in individuals with spinal trauma observed an incidence of concomitant spinal fracture in around 18%, with combined cervical and thoracic spine injuries being the most frequent (Kanna et al., 2016). These findings highlight the critical value of comprehensive whole spine imaging for identifying clinically occult fractures (Shimizu et al., 2025). While prior research has explored the value of cervical spine Magnetic Resonance Imaging (csMRI) following a cervical spine CT in geriatric trauma, findings were inconsistent and the yield of clinically significant injuries remains unclear (Westjem, 2021). Most recommendations advise against routine csMRI after a normal CT, with this guidance largely based on evidence from younger patients exposed to varied trauma mechanisms (Malhotra et al., 2018). Geriatric patients experiencing low-energy, ground-level falls may differ in both injury patterns and diagnostic needs, leaving the optimal imaging approach in this population unresolved (Tan et al., 2014).

Therefore, the aim of the study was to determine the prevalence of CSI following falls with head impact in patients aged 65 years and older and to identify factors associated with such injuries within the geriatric population. In addition, the study sought to evaluate the number needed to screen (NNS) to assess the incremental diagnostic yield of supplementary csMRI after cervical spine CT, as well as to identify concomitant spinal injuries with CT, including clinically silent cases. Age-stratified analyses were performed for patients aged ≥65 years and ≥80 years to investigate potential variations in diagnostic yield and risk profiles. In addition, a subgroup analysis of patients undergoing csMRI after CT evaluation was conducted to examine the prevalence and associated factors of CSI in this specific cohort.

## 2 Methods

### 2.1 Data collection

This retrospective study included geriatric patients aged 65 years or older presenting to the emergency department of a level I trauma center in Germany following a fall with subsequent head impact, all patients receiving at least both cranial CT (cCT) and cervical spine CT imaging between January 2020 and October 2025. In the emergency department, geriatric patients presenting with reduced vigilance, anticoagulant therapy or cognitive impairment due to dementia following a fall with head impact routinely underwent both cCT and cervical spine CT. Imaging was selectively extended based on clinical judgement to include polytrauma CT with whole-spine assessment. Trauma team activation in the resuscitation room followed a liberal protocol and was additionally triggered by predefined criteria, such as systolic blood pressure <100 mmHg, known or suspected traumatic brain injury with reduced Glasgow Coma Scale (GCS) score, involvement of two or more body regions or fractures of one or more long bones (Hranjec et al., 2012; Werman et al., 2011). In selected cases, complementary csMRI was performed to distinguish acute from chronic CSI, evaluate disco-ligamentous damage as well as in patients presenting with sensomotoric deficits, to inform further diagnostic assessment and guide therapeutic decision-making.

Collected data included demographic characteristics, injury mechanism, presence of sensomotoric deficits, cervical spine pain and CSI. CSI was classified into axial and subaxial trauma, with axial trauma defined as injuries involving C1–C2 and subaxial trauma defined as injuries involving C3–C7. Sensomotoric deficits were classified according to the American Spinal Injury Association (ASIA) impairment scale. Concomitant injuries were recorded, including intracerebral hemorrhage as well as facial, thoracic, pelvic, abdominal and upper or lower extremity trauma. Anticoagulant therapy, level of consciousness and pupillary responses were also documented, with anisocoria or delayed pupillary reaction considered pathological. Vigilance was assessed using the Glasgow Coma Scale, with scores ≤13 indicating impaired consciousness. Pre-existing conditions, including osteoporosis, ankylosing spinal disease, neurological comorbidities and histories of alcohol or nicotine abuse were recorded to provide a comprehensive characterization of patient baseline health. Clinical management and outcomes of CSI were recorded, including surgical versus conservative treatment and inpatient monitoring versus discharge. The study further assessed the prevalence of CSI and associated risk factors. Moreover, the NNS was determined to assess the additional diagnostic and therapeutic value of supplementary csMRI in patients with a CSI after initial CT, as well as to identify concomitant spinal injuries throughout the spine with CT, including those in clinically silent cases. Age-stratified analyses were also performed for patients aged ≥65 years and ≥80 years. Furthermore, a subgroup analysis was conducted on patients receiving additional csMRI to gain further insights. Overall, 90 geriatric patients were assessed and demographics, injury patterns and mechanisms, treatment approaches as well as associated factors were collected and analyzed.

### 2.2 Statistical analysis

The present study aimed to determine the prevalence of CSI following falls with head impact in patients aged ≥65 years and to identify factors associated with such injuries within the geriatric population. The analysis encompassed the evaluation of the NNS to determine the additional diagnostic and therapeutic value of supplementary csMRI in patients with a CSI after initial CT as well as to identify concomitant spinal injuries throughout the spine with CT, including those in clinically silent cases. Age-stratified analyses were performed for patients aged ≥65 years and ≥80 years to investigate potential variations in diagnostic yield and risk profiles. Furthermore, a subgroup analysis was conducted on patients receiving additional csMRI to gain further insights.

Statistical analyses were performed using SPSS (IBM SPSS Statistics, version 30.0.0, Zurich, Switzerland). Categorical variables were reported as frequencies and percentages, whereas continuous variables were summarized using means and standard deviations. Descriptive analyses enabled a comprehensive characterization of CSI in geriatric patients undergoing cranial and cervical spine CT imaging, as well as whole-spine CT and csMRI. Associations between patient characteristics and the occurrence of CSI following head trauma, as well as the utilization of csMRI in this population, were examined using odds ratios (ORs), applying a significance threshold of 5%. To identify independent predictors of CSI, multivariable logistic regression was performed employing a forward stepwise selection procedure. Variables were entered into the model according to their statistical significance (entry threshold: p < 0.05), beginning with the predictor demonstrating the strongest association with the outcome. The dependent variable was binary, indicating the presence or absence of CSI. Model fit and calibration were evaluated using the Hosmer–Lemeshow goodness-of-fit test. To reduce potential confounding and selection bias, the analysis incorporated a broad set of covariates, selected based on prior literature and theoretical considerations, including sociodemographic factors and health-related variables.

### 2.3 Ethical approval

The study was performed in accordance with the ethical principles of the Declaration of Helsinki and approved by the local ethics committee of the University of Regensburg (24-4010-104).

## 3 Theory

Geriatric trauma patients are highly susceptible to cervical spine injuries, even after low-energy mechanisms, due to age-related degeneration and reduced bone integrity. CT remains the primary diagnostic modality; however, the additional value of csMRI and whole-spine imaging is uncertain. While csMRI may detect disco-ligamentous injuries, its incremental clinical benefit appears limited in the absence of neurological deficits. In contrast, the recognized occurrence of noncontiguous spinal injuries supports a more comprehensive imaging strategy in selected cases. Thus, optimizing diagnostic pathways in geriatric trauma requires balancing diagnostic yield, resource utilization, and patient safety.

## 4 Results

A total of 2748 geriatric patients presenting with head trauma underwent both cranial and cervical spine CT upon arrival at the emergency department. The cohort had a mean age of 81.6 ± 7.8 years with 63.9% aged ≥ 80 years and 56.7% were female. Pre-existing conditions were notable, including dementia in 24.2%, cervical spinal canal stenosis in 6.9%, ankylosing spinal disorders in 1.5%, osteoporosis in 5.3% and neurological disorders in 19.1%, with 3.6% diagnosed with Morbus Parkinson. Anticoagulant therapy was recorded in 57.8% of the cohort. Upon admission, 15.8% exhibited impaired vigilance and 6.8% demonstrated abnormal pupillary responses. Regarding the mechanism of injury, 47.1% presented following a trip-and-fall, while 16.6% were triaged through the resuscitation room. Management in an intensive care unit (ICU) was necessary in 11.6% and in-hospital mortality was observed in 3.8% of cases (Table 1).

**Table 1:** demographic characteristics, pre-existing medical conditions and mechanisms of injury in geriatric patients with head trauma.

characteristics	N or average	% or SD
age	81.6	7.8
age ≥ 80 years	1756	63.9
female	1558	56.7

*pre-existing diseases*		
ankylosing spinal disease	42	1.5
osteoporosis	145	5.3
neurological disease	526	19.1
Morbus Parkinson	99	3.6
pre-existing cervical spinal canal stenosis	29	1.1
pre-existing dementia	666	24.2
cervical spinal canal stenosis	190	6.9
consumption of alcohol	180	6.6
nicotine abuse	90	3.3
anticoagulation	1588	57.8
impaired vigilance	435	15.8
pathological pupil reaction	188	6.8

*mechanism of injury*		
tripping fall	1294	47.1
nursing home fall	709	25.8
bicycle fall	112	4.1
traffic accident	258	9.4
stairs fall	266	9.6
walker-related fall	109	4.0
presentation via resuscitation room	456	16.6
intensive care unit monitoring	320	11.6
death	105	3.8

Following head trauma, 13.9% reported neck pain, while 1.5% exhibited sensomotoric deficits, most classified as ASIA D in 18 patients. Imaging was performed with 80.6% of patients undergoing combined cranial and cervical spine CT, whereas 19.4% received polytrauma CT imaging. Conventional radiographs (X-rays) of the cervical spine were obtained in 52 patients and csMRI was performed in 90 patients. In 6.1% of cases, csMRI provided additional diagnostic information, resulting in changes to therapeutic management not justified based on CT findings alone. A detailed overview of diagnostic findings by modality is provided in Table 2.

**Table 2:** Diagnostic findings in geriatric patients following head trauma.

characteristic	N	%
neck pain	383	13.9
sensomotoric deficit	40	1.5

ASIA Score		
A	4	0.1
B	4	0.1
C	14	0.6
D	18	0.7
E	2724	98.5

cervical spine X-ray	52	1.9

*CT-imaging*		
cCT & cervical CT	1422	51.7
cCT, cervical spine & midface CT	794	28.9
polytrauma-CT	532	19.4
cervical spine MRI	90	3.3

SCI was observed in 6.0%, with comparable frequencies of axial trauma involving C1 - C2 and subaxial trauma involving C3 - C7. Concomitant spinal trauma was observed in 6.6% and could involve any region of the spine, with thoracic fractures representing the predominant subtype. Among associated injuries, fractures of the lower extremities were the most frequent, affecting 11.8%, followed by thoracic trauma in 9.1%. Concomitant intracerebral hemorrhage occurred in 9.3%, with combined hemorrhage being the most prevalent. Conservative management of CSI was employed in 75.2% of cases, whereas 24.8% required surgical intervention, with dorsal instrumentation representing the most frequently performed technique in 63.4% of operative procedures. Additional cervical decompression was required in 0.8%. A comprehensive summary is provided in Table 3.

**Table 3:** distribution of injury and treatment strategies.

characteristics	N	%
cervical spine injury	165	6.0

*type of cervical spine injury*		
none	2582	94.0
axial trauma	75	2.7
subaxial trauma	77	2.8
both	1	0.5

concomitant spinal injury		
none	2567	93.4
cervicothoracic	24	0.8
thoracic	58	2.1
thoracolumbar	32	1.2
lumbar	43	1.6
sacral	24	0.9

*concomitant facial injury*		
none	971	35.3
soft tissue facial injuries	1245	45.3
facial skull fractures	142	5.2
soft tissue injuries & facial skull fractures	390	14.2
dental trauma	62	2.3

*type of cerebral hemorrhage*		
none	2492	90.7
intracranial hemorrhage	30	1.1
subarachnoid hemorrhage	56	2.0
epidural hematoma	3	0.1
subdural hematoma	46	1.7
combination	121	4.4

*concomitant injuries*		
thoracic trauma	251	9.1
pneumothorax	65	2.4
abdominal trauma	30	1.1
fracture upper extremity	325	11.8
fracture lower extremity	127	4.6
pelvis fracture	71	2.6

*treatment of cervical spine injury*		
conservative	124	75.2
operative	41	24.8
dorsal instrumentation	26	63.4
ventral instrumentation	12	29.3
dorsoventral instrumentation	3	7.3

cervical decompression	23	0.8

A binary logistic regression analysis was conducted using forward stepwise selection to identify significant predictors of the outcome variable CSI. In the final model, significant predictors included anticoagulation (B = 1.3, p = 0.02), pre-existing dementia (B = 1.6, p = 0.05), ankylosing spinal disease (B = 5.3; <0.001) and intracerebral hemorrhage (B = 1.4; p = 0.02). Model fit was assessed using the Hosmer–Lemeshow goodness-of-fit test, evaluating the agreement between observed and predicted event rates across risk deciles. The non-significant result (χ^2^ = 8.38, p = 0.39) indicates acceptable model calibration and supports the adequacy of the logistic regression model (Table 4).

**Table 4:** logistic regression results using forward stepwise selection.

predictor	regression coefficient (B)	95% CI	p-value
anticoagulation	1.3	0.5 – 1.9	0.02
pre-existing dementia	1.6	0.4 – 1.1	0.05
ankylosing spinal disease	5.3	2.4 – 11.6	<0.001
Intracerebral hemorrhage	1.4	0.4 - 1.6	0.02

Advanced age (≥80 years; OR 1.1, p = 0.03) and anticoagulation therapy (OR 1.2, p = 0.007) were significantly associated with an increased risk of CSI following head impact in geriatric patients. Additional patient-related risk factors included pre-existing dementia (OR 1.2, p = 0.01) as well as ankylosing spinal disease (OR 5.9, p < 0.001). Injury-related variables such as pneumothorax (OR 2.6, p = 0.007), thoracic (OR 2.9, p < 0.001) and abdominal trauma (OR 4.9, p < 0.001), fractures of the upper extremity (OR 1.9, p < 0.001) as well as concomitant intracerebral hemorrhage (OR 1.2, p = 0.05) were also identified as risk factors. Conversely, osteoporosis (OR 1.7, p = 0.33), underlying neurological disease (OR 0.1, p = 0.08), impaired vigilance (OR 0.9, p = 0.07) and female sex (OR 1.6, p = 0.006) were not significantly associated with cervical spine injury. A comprehensive overview of risk factors is provided in Table 5.

**Table 5:** factors associated with cervical spine injury in geriatric patients sustaining head trauma.

characteristics	OR	p-value	95% CI
female	1.3	0.12	0.9 – 1.8
**age ≥80 years**	**1.1**	**0.03**	**0.8**–**1.6**
**anticoagulation**	**1.2**	**0.007**	**0.7**–**1.6**
impaired vigilance	1.4	0.08	1.0 – 2.1
**pre-existing dementia**	**1.2**	**0.01**	**0.6**–**1.6**
alcohol consumption	1.0	0.85	0.5 – 1.8
**ankylosing disease**	**5.9**	**< 0.001**	**2.9**–**11.9**
osteoporosis	1.7	0.33	0.7 – 1.9
neurological disease	1.1	0.08	0.4 – 1.5
morbus parkinson	1.3	0.08	0.8 – 1.6
**concomitant intracranial bleeding**	**1.2**	**0.05**	**0.7**–**2.0**
**pneumothorax**	**2.6**	**0.007**	**1.3**–**5.4**
**thoracic trauma**	**2.9**	**< 0.001**	**2.0**–**4.4**
**abdominal trauma**	**4.9**	**< 0.001**	**2.1**–**11.7**
**fracture upper extremity**	**1.9**	**< 0.001**	**1.3**–**2.9**
fracture lower extremity	1.4	0.36	0.7 – 2.6
pelvis fracture	2.0	0.06	1.0 – 4.3

The NNS was calculated for the entire cohort of 2748 geriatric patients following head trauma and initially undergoing CT diagnostic. The NNS to detect additional SCI and to inform therapeutic decision-making using csMRI following an initial cervical spine CT was 54.9 among patients aged ≥65 years. In patients aged ≥80 years, the diagnostic yield of supplementary csMRI after an initially CT of the cervical spine was 45.3. When whole-spine CT imaging was performed following an initial cervical spine CT, the NNS to detect one additional injury at any spinal level was 29.4 in patients aged ≥65 years and 19.3 in patients aged ≥80 years. The NNS to identify an additional injury at any level of the spine in clinical silent patients, when whole-spine CT imaging was performed after an initial cervical spine CT was 33.3 for patients aged ≥65 years and 30.7 for those aged ≥80 years (Table 6).

**Table 6:** *number needed to screen (NNS) for yield cervical spine MRI, detection of concomitant spinal injuries and detection of concomitant spinal injuries in asymptomatic patients in a geriatric cohort* after head trauma.

characteristics	NNS
*Patients ≥ 65 years*	
Cervical spine MRI yield	54.9
Detection concomitant spinal injury	29.4
Detection concomitant spine injury in asymptomatic patients	33.3

*Patients ≥ 80 years*	
Cervical spine MRI yield	45.3
Detection concomitant spine injury	19.3
Detection concomitant spine injury in asymptomatic patients	30.7

Additionally, a subgroup analysis was performed on patients undergoing csMRI after the initial CT assessment. Hereby, 90 geriatric patients underwent additional csMRI after head impact and initial diagnostic with at least cCT and cervical spine CT, the mean age was 77.4 ± 7.2 years, with 60.0% aged ≥ 80 years and 66.0% being male. Relevant pre-existing conditions included cervical spinal canal stenosis in 14.4%, ankylosing spinal disease in 7.8% and osteoporosis in 3.3%. Neurological disease was present in 16.7% with Morbus Parkinson in 3.3% and pre-existing dementia in 7.8%. Sensomotoric deficits were observed in 32.2% with ASIA D being the most common, occurring in 16.7%. The leading mechanisms of injury were tripping falls with 47.1% followed by falls on stairs with 27.8%. Imaging predominantly consisted of polytrauma CT in 72.2%. More than half of the patients were initially managed via resuscitation room and 51.1% required ICU monitoring, with an overall mortality of 12.2% (Table 7).

**Table 7:** Demographics, comorbidities and clinical characteristics of patients with cervical spine MRI.

characteristics	N or average	% or SD
age	77.4	7.2
age ≥ 80 years	54	60.0
male	60	66.7

*pre-existing diseases*		
ankylosing spinal disease	7	7.8
osteoporosis	3	3.3
neurological disease	15	16.7
morbus parkinson	3	3.3
pre-existing spinal stenosis	29	1.1
pre-existing dementia	7	7.8
cervical spinal canal stenosis	13	14.4
anticoagulation	46	51.1
impaired vigilance	22	24.4
pathological pupil reaction	4	4.4

*mechanism of injury*		
tripping fall	1294	47.1
nursing home fall	5	5.6
bicycle fall	7	7.8
traffic accident	18	20.0
stairs fall	25	27.8
walker-related fall	1	1.1
sensomotoric deficit	29	32.2

ASIA Score		
A	2	2.2
B	5	5.6
C	7	7.8
D	15	16.7
E	73	81.1

*CT-imaging*		
cCT & cervical CT	16	17.8
cCT, cervical spine & midface CT	9	10.0
polytrauma-CT	65	72.2
presentation via resuscitation room	53	58.9
intensive care unit monitoring	46	51.1
death	11	12.2

In this subgroup analysis, CSI was observed in 71.1%, with subaxial trauma being the most common in 45.6%. Discoligamental cervical injuries occurred in 20.0%. Concomitant spinal injuries anywhere at the spine occurred in 41.1%, predominantly affecting the cervicothoracic region with 18.9%. Among concomitant injuries, thoracic trauma was the most frequent, affecting 33.3% followed by intracerebral hemorrhage in 20.0%. Treatment of CSI was predominantly conservative in 70.0%, while dorsal instrumentation was the most common type of operative procedure with 70.4%. Additional cervical decompression was performed in 21.1% (Table 8).

**Table 8:** Cervical spine injuries and their management in patients undergoing cervical spine MRI.

cervical spine injury	64	71.1
*type of cervical spine injury*		
none	26	28.9
axial trauma	13	14.4
subaxial trauma	41	45.6
both	10	11.1

discoligamental injury	18	20.0

concomitant spinal injury		
none	53	58.9
cervicothoracic	17	18.9
thoracic	5	5.6
thoracolumbar	10	11.1
lumbar	3	3.3
sacral	1	1.1

*concomitant facial injury*		
none	29	32.2
soft tissue facial injuries	40	44.4
facial skull fractures	6	6.7
soft tissue injuries & facial skull fractures	15	16.7

*type of cerebral hemorrhage*		
none	72	80.0
intracranial hemorrhage	3	3.3
subarachnoid hemorrhage	7	7.8
subdural hematoma	3	1.7
combination	5	5.6

*concomitant injuries*		
thoracic trauma	30	33.3
pneumothorax	6	6.9
abdominal trauma	5	5.6
fracture upper extremity	24	26.7
fracture lower extremity	6	6.7
pelvis fracture	2	2.2

*treatment of cervical spine injury*		
conservative	63	70.0
operative	27	30.0
dorsal instrumentation	19	70.4
ventral instrumentation	6	22.2
dorsoventral instrumentation	2	7.4

cervical decompression	19	21.1

Among patients undergoing csMRI, CSI was significantly associated with age ≥80 years (OR = 1.2, 95% CI 0.8–1.6, p = 0.04), pre-existing cervical spinal canal stenosis (OR = 1.2; p = 0.05), dementia (OR = 1.5, 95% CI 0.7–1.6, p = 0.01) and Morbus Parkinson (OR = 1.1, 95% CI 0.8–1.1, p = 0.01). Comorbidities such as ankylosing spinal disease (OR = 1.9, 95% CI 0.5–3.0, p = 0.34), osteoporosis (OR = 1.3, 95% CI 0.9–2.5, p = 0.08) and concomitant intracerebral hemorrhage (OR = 1.2, 95% CI 0.5–2.7, p = 0.51) were not significantly associated with CSI (Table 9).

**Table 9:** associations with cervical spine injury in a cohort with cervical spine MRI.

characteristics	OR	p-value	95% CI
female	1.1	0.52	0.7 – 2.6
**age ≥80 years**	**1.2**	**0.04**	**0.8**–**1.6**
anticoagulation	1.1	0.31	0.8 – 1.3
impaired vigilance	1.6	0.19	0.8 – 3.3
**pre-existing dementia**	**1.5**	**0.01**	**0.7**–**1.6**
ankylosing spinal disease	1.9	0.34	0.5 – 3.0
**cervical spinal canal stenosis**	**1.2**	**0.05**	**0.3**–**1.4**
osteoporosis	1.3	0.08	0.9 – 2.5
neurological disease	1.2	0.21	0.8 – 1.2
**morbus parkinson**	**1.1**	**0.01**	**0.8**–**1.1**
concomitant intracranial bleeding	1.2	0.51	0.5 – 2.7
thoracic trauma	1.3	0.32	0.7 – 2.4
abdominal trauma	1.7	0.24	0.7 – 3.9
**fracture upper extremity**	**1.5**	**0.05**	**0.8**–**1.2**
fracture lower extremity	1.8	0.26	0.6 – 2.3
pelvis fracture	1.1	0.23	0.9 – 1.3

## 5 Discussion

In this cohort of 2748 geriatric patients undergoing cervical spine CT and cCT imaging following head trauma, fall-related spine injuries imposed a substantial burden on emergency and hospital resources. Several risk factors for CSI were identified, with the most significant being age ≥80 years, presence of ankylosing spinal disease and pre-existing dementia. The low number needed to screen for detection of additional spinal injuries emphasizes the importance of cervical spine CT combined cCT, with the option to extend imaging to include the entire spine or csMRI in selected cases based on the clinical presentation, even after seemingly minor trauma.

### 5.1 Demographics, comorbidities and diagnostic characteristics

Rising life expectancy leaded to an increasing frequency of accidents and falls remain a major public health concern constituting the leading cause of injury in elderly people (Prabhakaran et al., 2020; Schindler et al., 2023). The use of CT in minor head injury remains debated, prompting the development of decision rules aimed at determining its necessity while considering both cost implications and radiation safety concerns (Squarza et al., 2019). Therefore, geriatrics presenting with fall-related injuries challenge clinicians to determine the appropriate imaging strategy (Engelbart et al., 2022). The decision is further complicated by the potential need for supplementary csMRI to provide additional diagnostic detail and the consideration of whole-spine imaging, even in asymptomatic patients, to detect clinically occult injuries (Shimizu et al., 2025; Kanna et al., 2016; Tan et al., 2014). In a cohort of 2748 geriatric patients undergoing cervical spine CT and cCT imaging following head injury, the mean age was 81.6 years with 57.8% receiving anticoagulation therapy, comparable to literature (Schindler et al., 2020, 2023; Squarza et al., 2019). Age ≥80 years (OR = 1.1; p = 0.03) and anticoagulation (OR = 1.2; p = 0.007) emerged as contributors. Advanced age was consistently identified as risk factor, reinforcing its importance in clinical context (Wang et al., 2013). Age-related physiological changes and the presence of comorbidities, contribute to both the susceptibility to injury and poorer outcomes observed in this population (Bank et al., 2018). Consistent with literature, dementia affected 24.2% and was identified as risk factors for CSI (OR = 1.2; p = 0.01) (GIOFFRÈ-FLORIO et al., 2018). Cognitive impairment can obscure symptom recognition, reduce the reliability of neurological examinations and delay the identification of potentially serious injuries, thereby posing a significant challenge to timely and accurate diagnostic evaluation (Lomoschitz et al., 2002; Higgs and Gilleard, 2017; Yee and Jain, 2025). In our study, the observed mortality rate was 3.8%, with 11.6% of patients subsequently requiring monitoring at the ICU, comparable with literature (Eryurt et al., 2023; Lee et al., 2022). Moreover, 1.5% were affected by ankylosing spinal disease and 5.3% had osteoporosis. Our findings indicate a lower occurrence of osteoporosis and ankylosing spinal disease than reported in literature (Jeanmonod and Varacallo, 2025; Squarza et al., 2019; Turchiaro et al., 2023). However, ankylosing spinal disease was assessed as risk factor for CSI (OR = 5.9; p < 0.001), whereas osteoporosis (OR = 1.7; p = 0.33) was not. In literature a substantial increase of risk for vertebrae fracture among osteoporotic patients was reported (Que et al., 2024; Fan et al., 2025; Sanfélix-Genovés et al., 2010). A possible explanation stems from the retrospective design of our analysis, as osteoporosis may not been documented in all patients, potentially underestimating its true prevalence and effect as risk factor for CSI. Moreover, a markedly elevated incidence of vertebral fractures in patients with ankylosing spinal disease was identified, underscoring the broader vulnerability of the spine due to structural rigidity and altered biomechanics (Zhang et al., 2017; Prieto-Alhambra et al., 2015). Sensomotoric deficits were observed in 1.5%, considerably lower than reported in a review of geriatric CSI, where partial neurological deficits were noted in 25.2% and complete deficits in 3.7% (Malik et al., 2008). Previous literature reports a substantially higher rate of sensomotoric deficits, including a greater prevalence of high-energy trauma. In this context, 40.2% of injuries were classified as high-energy, compared to 82.5% low-energy trauma in our study (Malik et al., 2008). Among the mechanism of injury, trip-related falls were most prevalent, accounting for 47.1%. Falls constitute the most common cause of CSI in elderly patients, with literature reporting over 60% of cases (Sethi et al., 2014; Jeanmonod and Varacallo, 2025; Malik et al., 2008). Clinical decision rules such as NEXUS and Canadian C-spine rule were proposed to guide cervical spine assessment, presenting important limitations in geriatrics (Stiell et al., 2001; Paykin et al., 2018; Tran et al., 2016; Jambhekar et al., 2018). A reliance on these decision rules alone is not recommended for geriatrics and practitioners frequently depend on individualized clinical judgment, despite the associated resource use and increased imaging costs (Engelbart et al., 2022; Tran et al., 2016; Jambhekar et al., 2018). Selective imaging strategies were proposed as effective method, in geriatrics using targeted CT approaches, essential for improving diagnostic accuracy, guiding treatment decisions and minimizing the risk of delayed intervention (Pearcy et al., 2023). In general, 80.6% of patients underwent both cCT and cervical spine CT scans, whereas 19.4% received polytrauma CT. To further enhance diagnostic evaluation, 3.3% underwent additional csMRI. Similar rates of cervical spine CT were reported in previous studies (Engelbart et al., 2022; Malik et al., 2008). In our study, 6.0% sustained CSI, with axial and subaxial trauma appearing at comparable frequencies. Literature indicates a high frequency of upper cervical spine trauma, particularly atlantoaxial fractures, in elderly patients due to reduced bone density, altered mechanisms of injury and degenerative changes affecting spinal biomechanics (Squarza et al., 2019). Moreover, literature indicates a higher risk of upper CSI in elderly patients following low-energy falls compared with those sustaining high-energy trauma (Lomoschitz et al., 2002). Conservative treatment was administered in 75.2% and in cases requiring surgical intervention, dorsal instrumentation was the predominant method, applied in 63.4%. This observation is in line with literature (Malik et al., 2008; Miller et al., 2010). Among patients with concomitant injuries, 6.6% sustained additional spinal trauma, most often involving the thoracic spine. Although previous studies documented rates about 20% for concomitant spinal injuries, thoracic injuries were still the predominant form of associated spinal injury (Shimizu et al., 2025; Lomoschitz et al., 2002). Among our cohort, fractures of the lower extremities were the most common associated injury, affecting 11.8%, followed by concomitant intracerebral hemorrhage in 9.3%. According to literature, lower limb trauma, represents a common consequence of fall-related injuries (Bergen, 2016; Engelbart et al., 2022).The literature reported comparable rates of intracerebral hemorrhage as concomitant injury (Engelbart et al., 2022). Trauma-related risk factors for CSI were thoracic (OR = 2.9; p < 0.001) and abdominal trauma (OR = 4.9; p < 0.001), lower extremity fractures (OR = 1.9; p < 0.001) and concomitant intracerebral hemorrhage (OR = 1.5; p = 0.03). With advancing age, progressive brain atrophy creates additional space for hemorrhage, potentially delaying symptoms (Yee and Jain, 2025). Such delays, combined with subsequent postponements in treatment initiation, contribute to the higher morbidity observed in geriatrics (Yee and Jain, 2025; Kirkpatrick and Pearson, 1978). To the best of our knowledge, our study is the first to evaluate NNS for the additional diagnostic yield provided by supplementary csMRI following CT, the NNS for detecting concomitant spinal fractures in patients with CSI as well as the NNS for concomitant spinal fractures in asymptomatic patients with CSI. The NNS for additional information from csMRI was 54.9 for patients aged ≥65 years and 45.3 for those aged ≥80 years. For detection of concomitant spinal injuries, the NNS was 29.4 for patients ≥65 years and 19.3 in patients aged ≥80 years, while among asymptomatic patients, the NNS was 33.3 for those ≥65 years and 30.7 for those ≥80 years. Multiple studies found limited benefit from routine csMRI in the absence of neurological deficits or clinical suspicion of instability (Malhotra et al., 2018; Tan et al., 2014). Malhotra et al., reported additional diagnostic information from csMRI in 20.9% of cases, yet only 6.0% of unstable injuries were detected and the therapeutic impact of these findings was limited (Malhotra et al., 2018). Consistent with literature, our results indicate no advantage of routine csMRI following an initial cervical spine CT (Kanna et al., 2016; Malhotra et al., 2018). The relatively high NNS for csMRI further supports the selective use in cases with clinical suspicion of injury (Tan et al., 2014). In contrast, the low NNS for detecting additional spinal injuries in patients with CSI for both age groups underscores the importance of thorough clinical examination. Concomitant spinal injuries are common in CSI, highlighting the need for comprehensive evaluation and in patients with limited clinical assessability or specific suspicion, generous consideration of supplemental imaging of the entire spine is warranted (Shimizu et al., 2025). In the subgroup undergoing additional csMRI, ankylosing spinal disease were prevalent at 7.8% and spinal canal stenosis at 14.4%. Sensomotoric deficits occurred more frequently at 32.2%, accompanied by a markedly higher rate of polytrauma CT at 72.2% and ICU monitoring at 51.1%, indicating a cohort with more severe injuries. CSI was identified in 71.1% with subaxial trauma accounting for 45.6%. Nevertheless, 70% of patients were managed conservatively.

Accordingly, timely cervical spine CT in combination with cCT should be routinely performed in all geriatric patients with head trauma, including low-energy injuries, while careful clinical assessment for additional spinal injuries is essential and in cases of clinical suspicion or limited assessability, imaging of the entire spine should be considered, whereas supplementary csMRI provides limited additional benefit and should be reserved for selected situations (Engelbart et al., 2022; Malhotra et al., 2018; Lomoschitz et al., 2002).

### 5.2 Limitations

Several limitations of this study warrant consideration. The retrospective design is susceptible to potential selection bias and limits the completeness of available data. Additionally, comorbidities such as neurological disorders and osteoporosis were documented retrospectively from the emergency department records and clinical findings might have been missed. Long-term clinical outcomes after discharge were not assessed, restricting insight into the enduring effects of the observed findings. Although logistic regression accounted for key confounders, residual confounding from unmeasured variables cannot be excluded. The exploratory analysis and the large number of variables considered may give rise to spurious associations. Results should therefore be interpreted cautiously and considered indicative rather than conclusive. Conducted at a single level I trauma center in Germany, the generalizability of results to other settings or healthcare systems remains limited, highlighting the need for multicentre validation.

## 6 Conclusion

CSI frequently occur in geriatric patients following fall-related head trauma, posing a significant challenge for healthcare systems. This study highlights the importance of routinely performing cervical spine CT alongside cCT when head imaging is indicated, facilitating prompt identification of CSI. Additional csMRI may be employed selectively in specific cases, while the low NNS for detecting concomitant spinal injuries reinforces the necessity of careful clinical assessment. In patients with clinical suspicion or limited evaluability, comprehensive imaging of the entire spine should be considered to prevent complications and enhance patient outcomes in the elderly trauma population.

## Informed consent

Not necessary.

## Ethics approval

Ethical Approval from the University Regensburg (24-4010-104).

## Data availability statement

Available on request.

## Authors’ contribution

The manuscript was created by JS and SL. JS, SL, MA, HP and MK performed the statistical analysis and designed the study. JS, SL, AH, JK, HP, MM, GN, BF, DP, VA, MK and SL conceived the study, helped to draft the manuscript and participated in its design. All authors read and approved the final manuscript.

## Funding

No funding was received.

## Declaration of competing interest

The authors declare that they have no known competing financial interests or personal relationships that could have appeared to influence the work reported in this paper.
